# CMR detects abnormal septal convexity into the left ventricle in preclinical hypertrophic cardiomyopathy

**DOI:** 10.1186/1532-429X-17-S1-P274

**Published:** 2015-02-03

**Authors:** Patricia Reant, Gabriella Captur, Mariana Mirabel, Arthur Nasis, Daniel Sado, Viviana Maestrini, Silvia Castelletti, Charlotte Manisty, Anna S Herrey, Daniel Jacoby, Maria T Tome Esteban, Perry M Elliott, William J McKenna, James Moon

**Affiliations:** University of Bordeaux, Bordeaux, France; Inherited Cardiac Diseases and CMR, Heart Hospital, London, UK; Georges Pompidou Hospital, Paris, France; UCLH, London, UK

## Background

Sarcomeric gene mutations are responsible for hypertrophic cardiomyopathy (HCM). In gene mutation carriers without significant left ventricular (LV) hypertrophy (G+LVH-), subtle abnormalities can exist as mitral valve elongation, crypts, markers of elevated LV systolic function, and abnormal apical trabeculation. Reverse curvature of the interventricular septum into the LV is a characteristic of G+LVH+ patients. We aimed to assess LV septal convexity into the LV in G+LVH-.

## Methods

Cardiovascular magnetic resonance was performed on 36 G+LVH- individuals (31±14 years, 33% men) with a pathogenic sarcomere mutation, and 36 sex and age-matched healthy volunteers (33±12 years, 33% men). Septal convexity (SCx) was measured in the apical four cavities view using a reference line joining the mid-septal wall at tricuspid valve insertion level and the apical right ventricular insertion. SCx was the maximal distance from this line to the LV endocardium border (A-B). The Figure depicts an example of SCx into LV in a G+LVH- (a) *vs*. matched control (b).

## Results

Mean septal convexity into LV was 5.0±2.5mm in G+LVH- *vs.* 1.6±2.4mm in controls (p≤0.0001). Compared to controls, G+LVH- individuals also had longer anterior mitral valve leaflet (23.5±3.0mm *vs.* 19.9±3.1mm, p≤0.0001), higher relative wall thickness (0.31±0.05 *vs.* 0.29±0.04, p≤0.05), higher ejection fraction (70.8±4.3% *vs.* 68.3±4.4%, p≤0.05), and smaller LV end-systolic volume index (21.4±4.4ml/m2 *vs.* 23.7±5.8ml/m2, p≤0.05). Other morphologic measurements of LV angles, sphericity index, and excentricity index were not significantly different between G+LVH- individuals and controls.

## Conclusions

Septal convexity into LV is an additional feature of preclinical HCM, occurring before the presence of any hypertrophy.

## Funding

Pr Moon is funded by the Higher Education Funding Council for England. Pr Elliott is funded by the British Heart Foundation, National Institute for Health Research (NIHR), and unrestricted educational grants Genzyme and Shire. Dr Captur is funded by the University College London (UCL), a Charlotte and Yule Bogue Research Fellowship, and a European Union Science and Technology Grant. Dr Reant and Mirabel are supported by the French Federation of Cardiology.

